# Effects of MDMA treatment and cessation on sexual behaviour and testicular functions in male sprague-dawley rats

**DOI:** 10.1186/s40360-026-01131-1

**Published:** 2026-04-11

**Authors:** O. O. Obembe, E. T. George, R. A. Mustapha, R. E. Akhigbe

**Affiliations:** 1 Department of Physiology, Osun State University, Osogbo, Osun State Nigeria; 2 Department of Physiology, Ladoke Akintola University of Technology, Ogbomoso, Oyo State Nigeria; 3 Reproductive Biology and Toxicology Research Laboratory, Osogbo, Osun State Nigeria

**Keywords:** MDMA, Sexual behaviour, Oxidative stress, Reproductive hormones, Inflammation

## Abstract

**Background:**

3,4-Methylenedioxymethamphetamine (MDMA) is a psychostimulant known for its social and empathogenic effects. However, its long-term impact on sexual performance and reproductive functions remain controversial.

**Objective:**

In this study, the effects of chronic MDMA exposure and subsequent withdrawal on sexual behaviour, reproductive hormones, sperm parameters, oxidative stress, systemic inflammation and testicular histology in male rats were investigated.

**Methods:**

Fifteen male rats were divided into three groups (*n* = 5 rats/group): control (distilled water-treated), MDMA-treated (100 mg/kg orally for 30 days), and MDMA-withdrawal (100 mg/kg of MDMA for 30 days followed by 30-day drug-free recovery). Sexual behaviour was assessed every ten days for 60 days. Epididymal sperm suspension analysis, serum levels of luteinizing hormone (LH), follicle-stimulating hormone (FSH), testosterone, markers of oxidative stress and inflammation interleukins 1beta, 6, and 10 (IL-1β, IL-6, IL-10), tumor necrosis factor-alpha (TNF-α) in serum and testes, and histological evaluations of testes were performed.

**Results:**

MDMA significantly enhanced sexual behavioural parameters (mount, intromission, and ejaculation frequencies) during exposure, but these declined with drug withdrawal. Sperm count and motility decreased following MDMA treatment, with partial recovery after withdrawal. Oxidative stress marker (malondialdehyde, MDA) and proinflammatory cytokines (IL-1β, IL-6, TNF-α) were significantly increased while antioxidant enzymes [superoxide dismutase (SOD), catalase, total thiol] were significantly decreased both in the serum and testes. MDMA also suppressed LH, FSH, and testosterone levels, while histological analysis showed reduced spermatid populations, which improved post-cessation.

**Conclusion:**

MDMA elicits prosexual effects that was accompanied by low epididymal sperm quality, oxidative stress, inflammation, and hormonal suppression during exposure. Nonetheless, partial recovery occurred following MDMA cessation, suggesting potential long-term reproductive risks.

## Introduction

Over the years, psychostimulants have been taken by humans to manage pain and distress, to induce euphoria or enhance the perception of experiences, and/or to stimulate behaviour in conditions of psychological or physiological inhibition. In recent times, these drugs have now been classified as drug of abuse, due to their potent activation of the brain’s reward circuitry, which induces euphoria, reinforces repeated use, and rapidly leads to dependence and compulsive behaviour [7, 43, 58]. Apart from their effect on the brain reward circuitry and their euphoric effects, these substances have been found to increase sexual activity; thus, they are now used as aphrodisiacs [36]. However, data existing in the literature on the aphrodisiac properties of psychostimulants are conflicting. These drugs modulate the release of catecholamine, neuropeptide, and nitric oxide, thus augment neural responses in hypothalamic or limbic structures to sexually arousing stimuli, in addition to the peripheral stimulation of appropriate sympathetic and parasympathetic responses that prepare the body for sexual interaction [22]. These drugs also enhance sexual arousal, desire, or pleasure, by enhancing the activation of excitatory systems for sexual behaviour (i.e. dopamine, norepinephrine, melanocortins, and oxytocin), or by supressing the activation of inhibitory systems that normally shut sexual response down (i.e. opioids, endocannabinoids, and serotonin) [1, 3, 8, 17]. Psychomotor stimulants increase sexual arousal or desire and enhance the intensity of sexual stimulation via a direct effect, such as the facilitation of erection or an increased sensory awareness that can amplify sexual stimulation and the intensity of orgasm [13, 30, 45, 49]. A common psychoactive drug is 3,4-Methylenedioxymethamphetamine.

3,4-Methylenedioxymethamphetamine (popularly called MDMA, ecstasy, sass, or molly) is a common psychoactive substance used primarily because of its empathogenic properties, that is, the drug increases feelings of sociability and closeness to others [25]. Studies have reported that ecstasy users describe inconsistent effects of MDMA on sexual desire. Specifically, most users report no desire for penetrative sex but only increased feelings of sensuality, and others reported that users of MDMA experienced either increased or decreased interest in initiating sexual activity [29, 34, 44, 60]. Some studies noted that MDMA increases sexual arousal in users, while others suggest that MDMA users are more likely than non-MDMA users to experience diminishing sexual interest [11, 26, 51]. However, it is important to note that the MDMA users in later studies had used the drug frequently and also reported use of other drugs, so it is unclear whether the alleged effects of MDMA on sexuality was affected by consumption patterns of MDMA, other drugs, or drug-drug interaction.

However, most of the studies on psychoactive drug use, including MDMA and sexual behavior are typically based on interviews of drug users and has mainly focused on sexual risk-taking. To this effect, the present study was designed to evaluate the effect of MDMA exposure and its withdrawal on sexual behavior, male sex hormones [luteinizing hormone (LH), follicle-stimulating hormone (FSH), and testosterone], and sperm quality. The involvement of oxidative stress and inflammation was also explored.

## Methodology

### Animal care and grouping

3,4-Methylenedioxymethamphetamine was obtained with prior approval of National Drug Law Enforcement Agency (NDLEA) Nigeria. Animals used were obtained from the Animal House, of The College of Health Sciences, Osun State University, Osogbo, Osun State, Nigeria. Fifteen male Sprague–Dawley rats weighing 106.78 ± 2.60 g were used for this study. In addition, fifteen female rats weighing 114.35 ± 2.53 g were used for the sexual behaviour study. The rats were housed in the Animal House of the Department of Physiology, Faculty of Basic Medical Sciences, Osun State University, Osogbo. The animals were kept in plastic cages and acclimatized for fourteen (14) days before commencement of the experimental procedure. Feed and water were made available *ad libitum*, except during behavioural trials. All the animal experiments and protocol were maintained according to the recommendations of the National Institute of Health (NIH, (2011) for laboratory animal care and use and reported in accordance with ARRIVE guidelines.

The male rats were randomly assigned into three experimental groups (5 rats per group). The control rats were vehicle-treated with 0.2 ml distilled water daily, while MDMA-treated rats received 100 mg/kg MDMA orally daily for 30 days and MDMA-treated+withdrawal group received 100 mg/kg MDMA orally daily for 30 days, followed by a 30-day drug-free recovery period. Based on previous studies, the experimental oral mean lethal dose (LD_50_) for MDMA in rats ranges from 160 mg/kg [54] to 325 mg/kg [21]. Additionally, Frith et al. [19] reported no fatalities in rats administered 100 mg/kg orally daily for 28 days. Considering these findings and observations in our plot study, a single dose of 100 mg/kg was used in this study. The dose used and the preparation method were as reported by Erhirhie et al. [18]. MDMA was administered and rats were tested during the day (i.e. during their normal sleep period) to model human use patterns, where MDMA is typically used by humans at night. Potential disruptions in the rats’ sleep pattern due to the psychostimulant were meant to mimic the effects in humans [14].

### Sexual behaviour determination

Sexual behaviour parameters were assessed at multiple time points viz. baseline (day 0, before MDMA administration) and days 10, 20, 30, 40, 50 and 60 by measuring sexual motivation (mount latency and mount frequency), sexual performance (intromission latency and intromission frequency), and sexual pleasure (ejaculation latency and ejaculation frequency). To minimize external influences, all tests were conducted under dim light in a dedicated testing room with no related animals in the same or adjacent rooms, thereby eliminating potential olfactory or auditory cues. Prior to experimentation, male rats were trained with sexually receptive females three times over four days, as recommended by Yakubu and Akanji [61]. Female receptivity was induced by sequential subcutaneous administration of oestradiol benzoate (10 µg/100 g body weight) 48 h before pairing and progesterone (0.5 mg/100 g body weight) 4 h before pairing, ensuring intense proceptivity and receptivity [4, 9]. During testing, each male was placed in a transparent cage and allowed a 30 min adaptation period before the receptive female was introduced. Preceptive and precopulatory behaviors were recorded using a camcorder. Adhering to standard procedures [10, 50], mount frequency (the number of mount attempts without intromission), intromission frequency (number of vaginal penetrations), and ejaculation frequency (number of semen expulsions characterized by rhythmic abdominal contractions) were recorded, along with the latencies to first mount, first intromission, and ejaculation.

### Sample collection and animal sacrifice

Twenty-four hours after the last treatment (day 30 of exposure and day 30 of withdrawal), the rats were euthanized with sodium pentobarbital (30 mg/kg) intraperitoneally. Blood samples were obtained by cardiac puncture and the testes and epididymis were excised.

### Sperm analysis

Sperm suspension was obtained from the left caudal epididymis using the diffusion method and analysed using a microscope (Olympus, Japan) as described by Obembe et al. [41]. The sperm suspension obtained was diluted with 0.5 mL Tris-buffer solution, and an aliquot of this solution was examined on a microscope slide (X400). Sperm count was determined using the newly improved Neubauer’s counting chamber (haemocytometer). The ruled part of the chamber was focused and the number of spermatozoa counted in five 16-square cells. Progressive sperm motility estimates were performed from three different fields in each sample and the mean of the three estimates was used as the final motility score. Sperm viability was assessed using the eosin/nigrosin staining technique. Viable sperm cells remained unstained, while the dead sperm cells were stained. Based on these observations, percentile viability was recorded. The sperm morphology examined and was considered abnormal when a physical anomaly was observed on the tail, neck, or head and was expressed as a percentage of morphologically normal sperm. Epididymal volume was estimated by immersing the epididymis in 5 mL of normal saline in a measuring cylinder. The volume of fluid displaced was recorded as the epididymal volume [39, 40].

### Determination of oxidative stress markers of the serum and testis

The blood collected was centrifuged at 3,000 rpm for 15 min and the serum obtained was refrigerated. Also, a fraction of the right testis was weighed and homogenized in phosphate buffer (pH 7.4). The homogenates were centrifuged at 10,000 rpm for 10 min in a cold centrifuge at 4 °C. The supernatant was carefully decanted and preserved at -20 °C. Oxidative stress markers viz. malondialdehyde (MDA), superoxide dismutase (SOD), catalase and total thiol were assessed in the serum and testicular homogenate obtained. MDA was determined using the method described by Stocks and Dormandy [55]. Briefly, 1 ml of the sample was combined with 2 ml of TCA-TBA-HCL, mixed thoroughly, and heated for 15 min. After cooling, the fluorescent precipitate was removed by centrifugation and absorbance of the sample determined at 535 nm. SOD activity was determined by the method of Misra and Fridovich [33]. The samples were diluted with distilled water in a ratio of 1:9. Aliquot of 0.2 ml of the diluted sample was added to the 2.5 ml of 0.05 M carbonate buffer. The increase in absorbance at 480 nm was monitored every 30 s for 150 s. Catalase activities were determined by the method described by Sinha [52], 0.1 ml of the sample was mixed with 1.0 ml of 0.01 M phosphate buffer (pH 7.4), and incubated for 10 min. After the reaction was stopped, the sample was centrifuged and the supernatant was used to quantify the amount of H_2_O_2_ to calculate catalase activity at 570 nm. Total thiol levels were measured using a spectrophotometric assay based on 2,2-dithiobisnitrobenzoic acid (DTNB; Ellman’s reagent), as described by Hu et al. [24]. Aliquot of the serum was mixed with Tris-EDTA buffer, followed by the addition of DTNB. The mixture was incubated at room temperature for 15 min, after which the absorbance of the yellow-colored complex was measured at 405 nm. A reagent blank (without sample) and a sample blank (with methanol replacing DTNB) were prepared in parallel. A glutathione (GSH) solution (50–100 µmol/L) was used as the calibrator, and total thiol concentrations were expressed in nmol/L.

### Measurements of inflammatory cytokines

Serum interleukin-1β (IL-1β) (Catalog number: 2865 A), interleukin-6 (IL-6) (Catalog number: MB-2899 A), interleukin-10 (IL-10) (Catalog number: MB-2912 A), and tumour necrosis factor-α (TNF-α) (Catalog number: MB-2868 A) were measured using respective ELISA kits (Nanjing Mornmed Medical Equipment Co., Ltd, China). The assay procedure involved setting up standards, samples, and control on a pre-coated plate, with a duplicate for accuracy. After 90 min incubation at 37 °C, biotin-labelled antibodies and HRP- streptavidin conjugate were added sequentially with washing in steps. TNB substrate was then added, incubated for 10 to 20 min, and stopped with 50 µl stop solution. The absorbance was read at 450 nm.

### Determination of hormonal levels

From the serum obtained, follicle-stimulating hormone (FSH), luteinizing hormone (LH), and testosterone were measured using their respective ELISA kits. The remaining fraction of the right testis was homogenized using chloroform-methanol buffer in a cold centrifuge at 4 °C. The post mitochondria fraction was decanted, and preserved at -20 °C, and thereafter, testicular testosterone was assayed. ELISA kits (Bio Inteco, UK) were used to assay FSH (Catalog number: B-1-R002332), LH (Catalog number: 202404002), and testosterone (Catalog number: 202404001). This procedure involved setting up standard, pilot samples and control wells on the pre-coated plates, with a duplicate for each. After washing 50 µl of standards, samples, and control were added followed by 50 µl of biotin-labelled antibody solution. The plates were incubated at 37 °C for 45 min, washed three times, and incubated with HRP- streptavidin conjugate for 30 min. After 5 washes 90 µl of TNB substrate was added and the plate was incubated in the dark for 10 to 20 min, the reaction was terminated with 50 µl of stop solution, and the absorbance was read at 450 nm using a microplate reader.

### Histological examination

After harvesting the left testis, tissues were fixed in Bouin’s solution. It was later embedded in paraffin and 5 μm thick sections were prepared and stained with Hematoxylin and Eosin using standard procedures. The slides were viewed under a light microscope (Celestron LCD digital microscope, USA, model 44348) and photomicrographs of a representative from the five testicular tissue per group were taken at x 200 magnification.

### Statistical analysis

All the values are expressed as mean ± standard error of mean (SEM) of five replicates. Analysis of data was done using GraphPad Prism version 8.0.2 for Windows. Differences between groups were analysed by one-way ANOVA followed by *Bonferoni post-hoc test*. Differences were considered significant when *P* < 0.05.

## Results

### Effect of MDMA on sexual behaviours

**Fig. 1:**
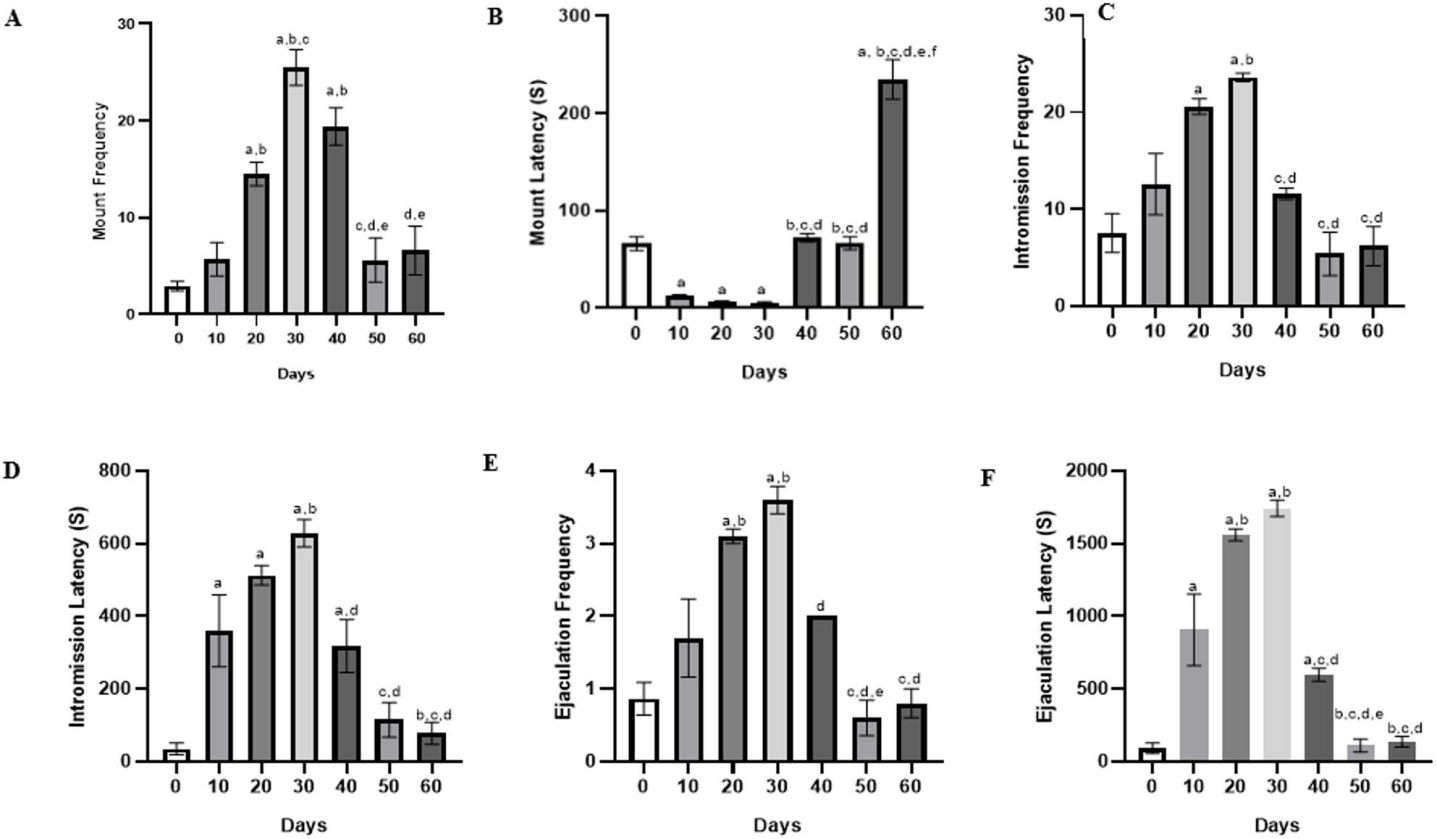
Effect of MDMA on sexual behaviour; (**a**) mount frequency, (**b**) mount latency, (**c**) intromission frequency (**d**) intromission latency, (**e**) ejaculation frequency and (**f**) ejaculation latency. Values are *Mean ± SEM*, ^a−^ significant difference from day 0, ^b−^ significant difference from day 10, ^c−^ significant difference from day 20, ^d−^ significant difference from day 30, ^e−^ significant difference from day 40 and ^f−^ significant difference from day 60

Mount frequency (MF) increased incrementally in MDMA treated rats, with significant increase on days 20 and 30 of treatment when compared with the baseline. After MDMA withdrawal, MF declined in succeeding days, with values significantly higher than the baseline. MF on days 50 and 60 were however comparable to that of day 10 (Fig. 1a). Mount latency (ML) significantly decreased by day 10 and continued to decline on days 20 and 30 compared to baseline. A significant increase occurred on day 40, with ML returning to baseline by day 50. By day 60, ML rose significantly above all previous values (Fig. 1b). Intromission frequency (IF) increased significantly by day 20 and peaked on day 30 compared to earlier time points. A marked decline occurred on day 40, continuing through days 50 and 60, with values falling below the baseline by day 60 (Fig. 1c). Intromission latency (IL) increased significantly by day 10, with further elevation on days 20 and 30 compared to baseline. Following MDMA withdrawal, IL declined significantly from day 40 onwards, though values remained above baseline throughout the study (Fig. 1d). Ejaculation frequency (EF) increased significantly by day 20 and peaked on day 30 compared to earlier timepoints. A marked decline was observed from day 40 onwards, with EF returning to baseline by days 50 and 60 (Fig. 1e). Ejaculation latency (EL) increased significantly by day 10 and peaked on day 30 compared to earlier days. A marked decline occurred by day 40, with further reduction on day 50. EL remained low and comparable on day 60 (Fig. 1f).

### Effects of MDMA on sperm parameters

**Fig. 2:**
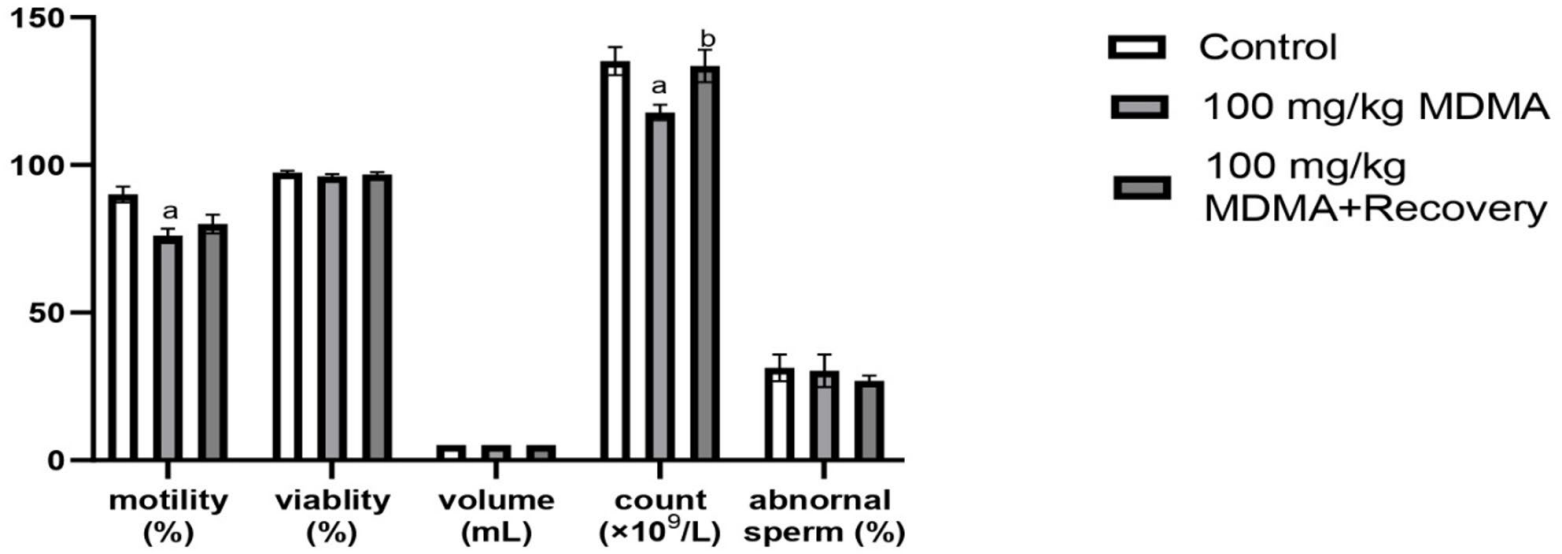
Effect of MDMA on sperm parameters. Values are *Mean ± SEM*, ^a−^ significant difference from the control group, ^b−^ significant difference from the 100 mg/kg MDMA

MDMA administration significantly reduced sperm motility and sperm count in treated rats when compared with the control. After 30-day recovery period, sperm motility remained low, while partial recovery of sperm count was observed but both values remained below when compared with the control. Abnormal sperm morphology slightly increased and persisted post-recovery, while sperm viability and semen volume were not affected (Fig. 2).

### Effect of MDMA on oxidative stress markers, and inflammatory cytokines

**Fig. 3:**
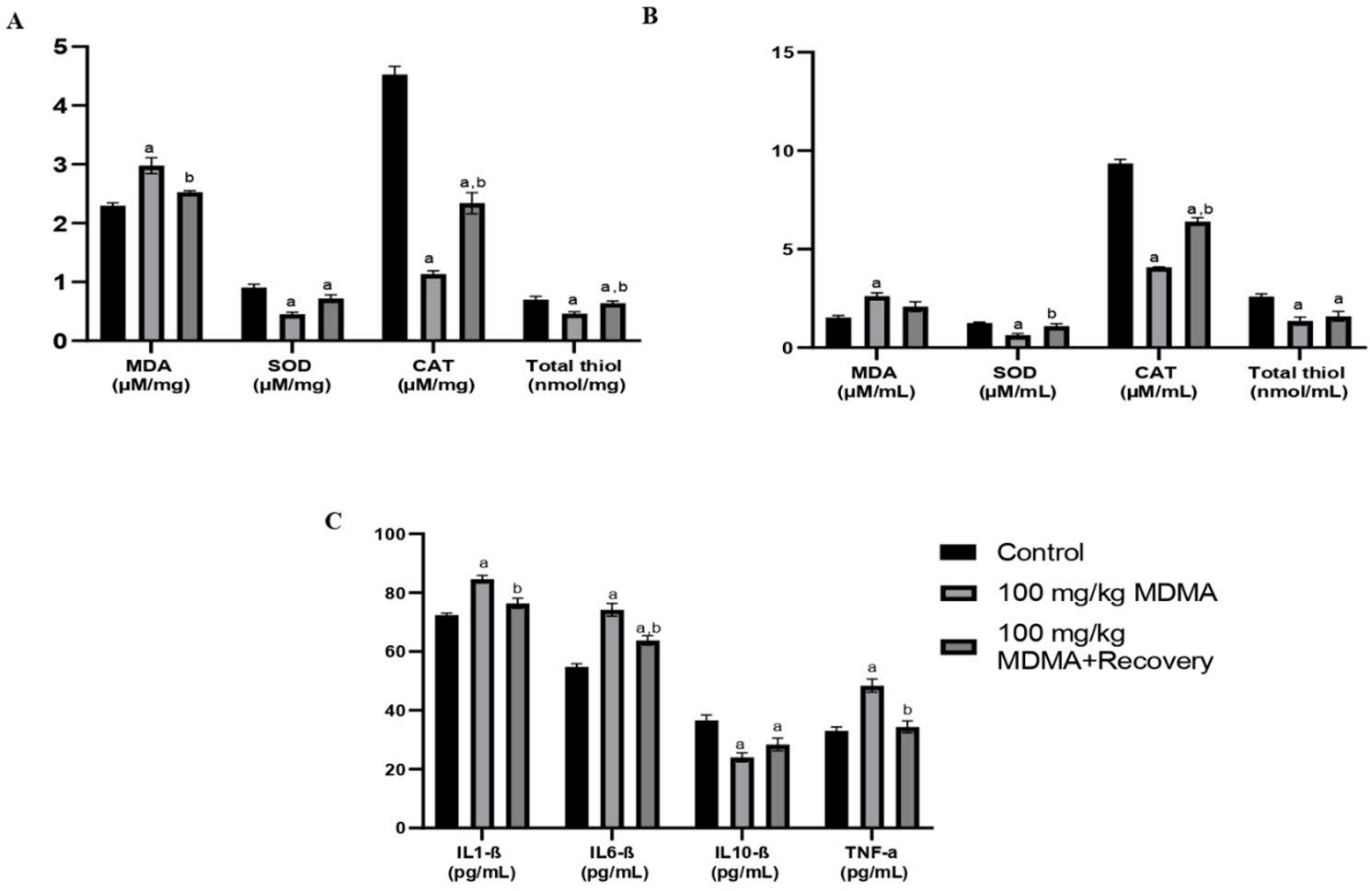
Effect of MDMA on markers of oxidative stress and inflammation; (**a**) testicular oxidative stress marker, (**b**) serum oxidative stress marker, (**c**) serum inflammatory marker. Values are *Mean ± SEM*, ^a−^ significant difference from the control group, ^b−^ significant difference from the 100 mg/kg MDMA

MDMA treatment for 30 days significantly increased serum and testicular MDA levels and elevated IL-1β, IL-6, and TNF-α, while reducing SOD, CAT, total thiols, and IL-10 compared with corresponding controls. After a 30-day recovery, alterations in the levels of the oxidative markers persisted, except MDA levels which was almost restored to the control levels. Also, IL-1β and TNF-α showed significant improvement when compared with the control after 30-day recovery, but IL-6 and IL-10 levels remained significantly altered relative to controls (Fig. 3a–c).

### Effect of MDMA on reproductive hormones

**Fig. 4:**
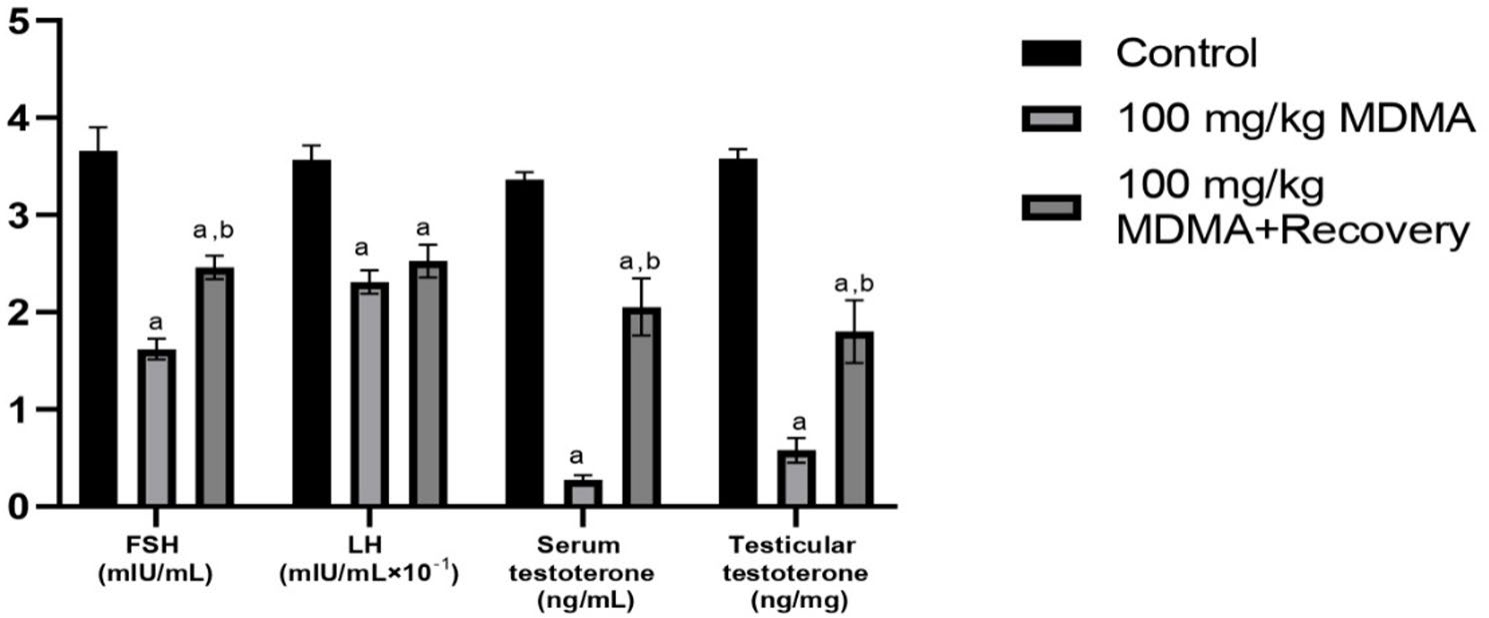
Effect of MDMA on serum and testicular reproductive hormones. Values are *Mean ± SEM*, ^a−^ significant difference from the control group, ^b−^ significant difference from the 100 mg/kg MDMA

Serum levels of FSH, LH, and testosterone, as well as testicular testosterone concentration were significantly reduced in rats treated with MDMA when compared with control. Although a 30-day recovery period led to significant improvements in the testosterone levels, the gonadotropic hormones remained markedly low, and the testosterone remained below control values (Fig. 4).

### Effect of MDMA on testicular histoarchitecture

**Fig. 5:**
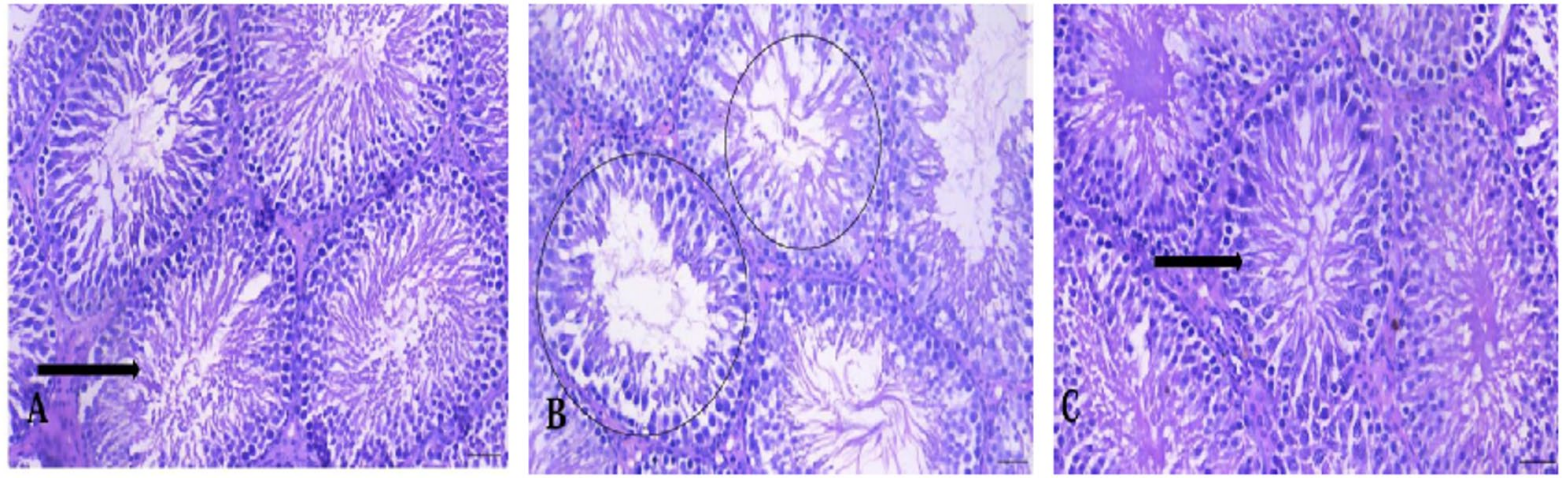
Effect of MDMA on testicular histoarchitecture. Group (**A**), shows normal testicular tissue that is composed of numerous seminiferous tubules displaying sequential maturation of the spermatogenic series (black thick arrow). In the group administered MDMA (**B**), shows testicular tissue with numerous seminiferous tubules which scanty sperm cells in the tubular lumen (black circle). Following a 30-day recovery period (**C**), the testicular tissue shows sequential maturation of the spermatogenic series (black arrow)

In the control group, testicular tissue showed normal seminiferous tubules with sequential maturation of the spermatogenic series and no abnormalities. MDMA-treated rats showed scanty sperm cells in the tubular lumen. After a 30-day recovery, tissue displayed normal maturation without adverse features (Fig. 5).

## Discussion

In this study, the effects of MDMA and its withdrawal on sexual behaviour, reproductive hormones, sperm parameters, oxidative stress, inflammatory cytokines, and testicular histoarchitecture in male Sprague-Dawley rats were investigated. Our findings revealed that MDMA administration significantly enhanced sexual performance indices (mount frequency, intromission frequency, ejaculation frequency) during therapy. However, these parameters markedly reversed to near the baseline values post-withdrawal, except the mount latency which increased tremendously. It is worth noting that MDMA treatment led to significant oxidative damage and inflammation in testicular tissue and serum, which was associated with suppressed reproductive hormones. These culminated in impairment of sperm parameters and altered sperm morphology. These results suggest that transient enhancement in sexual activity observed in this study as earlier reported in the literature [12, 56] is not mediated by the endocrine pituitary-gonadal axis but likely due to central neurotransmitter stimulation [31, 64]. Therefore, the dual nature of MDMA, exhibiting both pro-sexual and toxic activities.

The observed increase in sexual behaviour parameters such as mount frequency and intromission frequency during MDMA exposure may be attributed to its effects on neurochemical interactions. One of the well-known mechanisms of action of MDMA is its action on the vesicular monoamine transporter (VMAT2), which causes increase in synaptic availability of serotonin, dopamine, norepinephrine, and oxytocin [27], which are important neurotransmitters that mediate sexual behaviour [5]. Dopamine particularly plays a key role in sexual motivation and reward, and its documented elevation during MDMA exposure [42] likely accounts for increased mounting and intromission. MDMA elevates dopamine release in the prefrontal cortex by reversing the function of dopamine transporter [46]. The released dopamine activates D1 receptors, resulting in the stimulation of protein kinase A (PKA) activity, leading to dopamine- and cAMP-regulated neuronal phosphoprotein (DARPP-32) phosphorylation and increased cyclic AMP-responsive element-binding protein (CREB) phosphorylation, which upregulates c-fos expression in circuits that govern sexual motivation [20, 47]. Together, these mechanisms possibly mediate the observed increased mount and intromission frequencies, and decreased mount latency during active MDMA exposure.

MDMA has been documented to affect serotonin transporter, and therefore released serotonin activates both 5-HT2A and 5-HT2C receptors [16]. Activation of these receptors enhance glutaminergic neurons excitability, leading to increased output from the medial prefrontal cortex to regions such as the nucleus accumbens and hypothalamus, strengthening inhibitory control pathways [37] that are associated with extended ejaculation latency. Another probable reason for the changes in sexual behaviour as seen in our study may be due to the effect of MDMA on contextual processing and sexual memory. In this context 5-HT1A receptor activation modulates the firing patterns of pyramidal neurons, thereby altering the encoding of sexual environmental cues [48]. Also, the effect of MDMA on the protein brain-derived neurotrophic factor signalling further strengthens synaptic connections and supports the formation of sexual memories [35]. These adaptations can lead to context-dependent changes in mount frequency and a progressive decrease in mount latency over time. Despite the reduction in testosterone levels during MDMA exposure, sexual activity was still increased. This is possibly due to the dominant role of central dopaminergic and oxytocinergic stimulation overriding peripheral androgenic inputs, especially during acute exposure. Serotonin, which generally inhibits sexual behaviour, is rapidly metabolized post-release, thereby allowing the pro-sexual effects of dopamine and norepinephrine to predominate [57]. However, after MDMA withdrawal, sexual behaviour declined sharply, correlating with obviously decreased central neurotransmitter stimulation and persistent peripheral hormonal suppression. Thus, the transient enhancement of sexual activity during MDMA exposure likely reflects central excitatory neurotransmission rather than androgen-dependent processes.

MDMA exposure significantly increased the levels of malondialdehyde and decreased antioxidant levels (SOD, CAT, total thiol), both in serum and testis, indicating oxidative stress. This redox imbalance is a key contributor to testicular dysfunction [2]. MDMA metabolism produces reactive oxygen species (ROS) and quinone metabolites which overwhelm antioxidant defences, leading to lipid peroxidation and cellular damage [53]. The testes, with high polyunsaturated fatty acids and active steroidogenesis, are particularly vulnerable to oxidative injury; hence, the testes are highly susceptible to the oxidative damage from MDMA metabolites. Furthermore, MDMA upregulated pro-inflammatory cytokines (IL-1β, IL-6, TNF-α) and suppressed the anti-inflammatory cytokine (IL-10). This cytokine imbalance promotes leukocyte infiltration and cytokine-mediated disruption of Sertoli and Leydig cell function, impairing spermatogenesis and testosterone synthesis [23]. Elevated oxidative stress and inflammation synergistically damage germ cells, reduce sperm quality, and disrupt endocrine signalling as observed in the present study. These findings align with earlier reports [6, 38] that documented the oxidative and inflammatory effects of MDMA in the testis. Notably, although some antioxidant and cytokine parameters improved post-withdrawal, recovery was incomplete, indicating that MDMA-induced testicular injury persists even after drug cessation.

The significant reductions in FSH, LH, and testosterone observed post-MDMA exposure reflect hypothalamic-pituitary-gonadal (HPG) axis suppression. MDMA likely interferes with gonadotropin-releasing hormone (GnRH) secretion through serotonergic overactivation or stress-induced hypothalamic dysfunction [15]. Additionally, testicular oxidative stress may impair Leydig cell steroidogenesis, further reducing testosterone levels [63]. The downstream effects of hormonal suppression were evident in the sperm profile, resulting in significantly decreased sperm count and sperm motility, while abnormal morphology increased. Spermatozoa, just like the testis, are highly sensitive to ROS, which damage membranes, DNA, and mitochondria, thereby impairing motility and viability [59]. The partial recovery of hormone levels and sperm parameters after 30 days of MDMA withdrawal suggests possible reversible injury but with a likelihood for infertility. These outcomes parallel studies by Yamamoto and Raudensky [62] and Zaki and Abdel-Kawy [63], which demonstrated MDMA-induced reproductive toxicity through neuroendocrine and oxidative mechanisms. The link between inflammatory cytokines and sperm quality decline further supports the role of immune-mediated testicular disruption [28]. Therefore, systemic inflammation and oxidative stress are mechanistically interconnected with hormonal disruption and testicular dysfunctions.

The trajectory from initial pro-sexual stimulation to eventual reproductive toxicity shows the biphasic effect of MDMA on male reproductive function. During exposure, sexual activity is enhanced via dopaminergic and oxytocinergic pathways, despite low testosterone. However, these events are accompanied by neuroendocrine suppression, oxidative stress, inflammation, and testicular damage. Furthermore, the histological evidence of reduced germ cells confirms germ cell depletion due to cellular injury. This cascade of neurochemical excitation, redox imbalance, immune dysregulation, and endocrine disruption aligns with previous studies on methamphetamine and other psychostimulants [32]. The interaction between central neurotransmission and peripheral testicular function explains the increased sexual behaviour during exposure and its decline post-withdrawal. The persistent testicular injury despite withdrawal suggests long-term neuroendocrine reprogramming.

Although the study provides convincing data, there are some limitations. First, since the study exposed rats to MDMA for 30 rats before withdrawal, it is important to know if MDMA cessation after prolonged use will cause a similar effect. Also, the very small sample size of five animals per group used in this study may limit the statistical power of the study and restrict the generalizability of the findings. Increasing the sample size would improve the robustness and reliability of the results, thereby strengthening the overall conclusions of the study. Furthermore, the study was conducted in a Sprague–Dawley rat model; thus, extrapolating the results of this study to human should be with utmost caution.

## Conclusion

MDMA exerts pro-sexual effect but paradoxically causes endocrine disruptions, oxidative stress, and inflammation, which only partially resolve after cessation. Future research should explore dose-dependent effects of MDMA on both male and female reproductive physiology, using multiple exposure designs to reflect recreational and chronic use.

## Data Availability

The data used to support the findings of the present study are available from the corresponding author upon request.
